# Colorectal cancer survivors’ beliefs on nutrition and cancer; correlates with nutritional information provision

**DOI:** 10.1007/s00520-019-04934-7

**Published:** 2019-06-21

**Authors:** Merel R. van Veen, Floortje Mols, Lian Smeets, Ellen Kampman, Sandra Beijer

**Affiliations:** 1 Division of Human Nutrition and Health, Wageningen University, P.O. Box 17, 6700 AA Wageningen, The Netherlands; 2 Department of Research & Development Netherlands Comprehensive Cancer Organisation (IKNL), Utrecht, The Netherlands; 3 CoRPS-Center of Research on Psychology in Somatic diseases, Department of Medical and Clinical Psychology, Tilburg University, Tilburg, The Netherlands

**Keywords:** Colorectal cancer survivor, Nutrition, Information provision, Health professionals

## Abstract

**Purpose:**

To investigate CRC survivors’ beliefs on nutrition and cancer and the association with nutritional information provision by (kind and number) of health professionals and to inquire about foods that CRC survivors believed either had a positive or negative influence on their cancer.

**Methods:**

A total of 326 CRC survivors of an ongoing prospective cohort study filled out questionnaires 1 month after surgery on whether they had received nutritional information from health professionals. Also, their beliefs that nutrition influences (1) feelings of well-being, (2) complaints after treatment, (3) recovery and (4) cancer recurrence were investigated. Prevalence ratios were calculated (using Cox proportional hazard regression analysis) to study associations between information provision and the four beliefs adjusted for age, gender and cancer stage.

**Results:**

Sixty-two percent of respondents received information about nutrition from one or more health professionals. Most respondents who received information strongly believe nutrition influences feelings of well-being (59%) and recovery after cancer (62%). Compared with those who did not receive information, respondents who received information from three professionals showed the strongest beliefs on the influence of nutrition on complaints after treatment (PR 3.4; 95% CI 1.6–7.4), recovery after treatment (PR 2.0; 95% CI 1.2–3.3) and recurrence (PR 2.8; 95% CI 1.3–6.2).

**Conclusion:**

Nutritional information provision by health professionals positively influences the beliefs of CRC survivors on the influence of nutrition on cancer outcomes: stronger beliefs occur when respondents received information from three health professionals.

## Introduction

Colorectal cancer (CRC) is the third most common cancer worldwide [1]. Due to the ageing of the population, implementation of screening programs and ongoing advancements in treatment, incidence and survival rates of CRC have increased over the past years [2], resulting in an increase in CRC survivors [3]. A person is characterized as a cancer survivor from the moment of diagnosis until the person deceases [4].

Before, during and after treatment, CRC survivors often suffer from nutrition-related symptoms such as changes in defecation, intestinal cramps, lack of appetite and unintended weight gain or weight loss [5, 6], which have a negative impact on quality of life [7, 8]. Dietary guidelines to alleviate these symptoms are available [6, 7, 9] and it is important that CRC survivors are able to access and follow this information in order to change their diet to improve their quality of life.

In addition, prospective cohort studies in cancer survivors have shown that higher adherence to the World Cancer Research Fund/American Institute for Cancer Research (WCRF/AICR) guidelines for cancer prevention [10] is associated with lower mortality in CRC survivors [11, 12] and better health-related quality of life [13, 14]. A healthy diet low in fat, meat and refined grains, combined with a high level of physical activity, has been shown to be associated with lower recurrence and mortality rates and a decreased risk of comorbid conditions in cancer survivors in general [11, 13, 15] and specifically in CRC survivors [16, 17].

Although the diagnosis of cancer is seen as a teachable moment [18], an event which presents a good opportunity for learning something about a particular aspect of life, only a minority of CRC survivors change their diet after diagnosis. Results from a cross-sectional study in 1458 CRC survivors showed that only 36% of CRC survivors reported that they had changed their diet after diagnosis [19]. Another cross-sectional study in 1196 CRC survivors found that 32% of CRC survivors intended to adopt a healthier diet; however, only 25% changed their diet after diagnosis of CRC. This study also found that CRC survivors’ adherence to the WCRF/AICR guidelines for cancer prevention was low, with 9% adhering to the recommendation for fruit and vegetable intake, and 12% adhering to more than six out of eight recommendations [20].

It is unknown why only few CRC survivors change their diet after diagnosis and why low adherence to healthy lifestyle recommendations persists even after a cancer diagnosis. One hypothesis is that CRC survivors think they already follow a healthy diet, as was seen by Anderson et al., where cancer survivors were sceptical that poor diet caused cancer, because people believed their diets were healthy before onset [21]. Another hypothesis is that CRC survivors do not believe nutrition can have an effect on cancer and cancer outcomes, possibly due to poor nutritional information provision. Two studies among CRC survivors found that only 17–19% of survivors received advice on nutrition or supplement use [17, 18].

The aim of the current study is (1) to investigate CRC survivors’ beliefs on nutrition and cancer and their association with information provision on nutrition by health professionals, (2) to investigate the association between the kind and number of health professionals and the strength of beliefs and (3) to categorize foods that CRC survivors believed either had a positive or negative influence on their cancer.

## Methods

### Study design and study population

The PROCORE study, started in 2016, is a prospective population based study, in which newly diagnosed CRC survivors are recruited before the start of treatment and followed via the PROFILES-registry (Patient Reported Outcomes Following Initial treatment and Long term Evaluation of Survivorship) [22] until 2 years after diagnosis. Ethical approval for the study was obtained from the certified Medical Ethics Committee of Medical Research Ethics Committees United (approval number NL51119.060.14). All respondents gave informed consent. Data from this longitudinal study will be available online for non-commercial scientific research, subject to study question, privacy and confidentiality restrictions and registration (www.profilesregistry.nl). For this specific paper, data from baseline (e.g. pre-treatment) and data from 4 weeks after surgery were used.

Respondents were recruited from four Dutch hospitals: the Catharina Hospital in Eindhoven, Maxima Medical Centre in Veldhoven, Elkerliek Hospital in Helmond and Elisabeth-TweeSteden Hospital in Tilburg. Inclusion criteria were the diagnosis of CRC stage I–IV and being 18 years or older. Exclusion criteria were ever being diagnosed with a different carcinoma, except for basal cell carcinoma of the skin; having cognitive limitations or being unable to read or write Dutch, which did not allow them to independently fill out a questionnaire.

### Data collection

CRC patients were identified by the research nurses or case managers (depending on hospital). They informed patients about the study and asked them to participate, before start of the treatment. Patients received an information package from the nurse or case manager, including a letter, a patient information leaflet, an informed consent form and a questionnaire. The informed consent and questionnaire could be send back to the PROFILES registry in two separate envelopes. Patients could indicate if they wanted to receive the follow-up questionnaires in digital form via the PROFILES registry, or on paper. Patients were reassured that nonparticipation had no consequences for their follow-up care or treatment.

A total of 595 people recently diagnosed with CRC were invited to participate in the PROCORE study. Of those, 403 people filled out the baseline questionnaire. Of these respondents, 344 underwent surgery for their CRC and were sent the second questionnaire, which was filled out by 326 survivors.

#### Questionnaires

For the current research question, data was obtained from the baseline survey and from the survey 4 weeks after surgery. The baseline survey before surgery consisted of self-designed questions on general characteristics, including age, height, usual body weight, body weight at the moment of diagnosis, highest level of education (elementary school, high school, vocational education, bachelor degree), smoking (current smoker, non-smoker, former smoker) and on alcohol consumption (current drinker (with mean intake), former drinker, never). The questionnaire 4 weeks after surgery consisted of self-designed questions on nutritional information provision by health professionals, and patients’ beliefs that nutrition influences feelings of well-being, complaints after treatment, recovery and cancer recurrence. Depending on the question, answers could be indicated on an ordinal Likert scale with four options (not at all, a little, some, a lot), on a scale from 0 to 10, or could be answered with ‘yes’ or ‘no’. Two open-ended questions were included asking the respondents to mention foods, diets or supplements they believed to either positively or negatively affect cancer.

Patients’ sociodemographic and clinical information was retrieved from the Netherlands Cancer Registry (NCR), including gender, diagnosis and tumour staging.

### Data analyses

Personal and disease-related characteristics, percentage of respondents who received nutritional information and percentage of respondents who strong, intermediate or do not believe that nutrition can influence feelings of well-being, complaints after treatment, recovery after treatment and cancer recurrence were described for the total group of respondents (*n* = 326; Tables 1 and 2). To investigate whether strong believers on one belief are also strong believers on the other beliefs, the characteristics of the study population were also split out by the belief of the influence of nutrition on well-being using the following categories: no belief of an influence (score 0–2, *n* = 63), an intermediate belief (score 3–6, *n* = 78) and a strong belief (score 7–10, *n* = 171). Differences between the ‘no belief’, ‘intermediate belief’ and ‘strong belief’ groups were analysed using Chi-square test.

**Table 1:** General characteristics of the total group of respondents (*n* = 326) and split in three groups based on the score for “belief that nutrition influences feelings of well-being”

	Respondents (*n* = 326)	Score 0–2 No belief influence (*n* = 63)	Score 3–6 Intermediate belief influence (*n* = 78)	Score 7–10 Strong belief influence (*n* = 171)
	*n* (%)	*n* (%)	*n* (%)	*n* (%)
Gender				
Male	198 (61)	42 (67)	41 (53)	107 (63)
Female	128 (39)	21 (33)	37 (47)	64 (37)
Age Years (mean ± SD)	67.2 (8.9)	68.8 (8.4)	66.9 (9.2)	66.2 (8.8)
< 70 years	191 (59)	32 (51)	43 (55)	112 (65)
≥ 70 years	135 (41)	31 (49)	35 (45)	59 (35)
Highest level of education*				
Elementary school/High school	108 (33)	28 (44)	30 (38)	43 (25)
Vocational education	130 (40)	20 (32)	30 (38)	76 (44)
Bachelor degree or higher	81 (25)	11 (17)	18 (23)	50 (29)
Missing	7 (2)	4 (6)	0 (0)	2 (1)
Smoking				
Current smoker	31 (10)	6 (10)	5 (6)	17 (10)
Former smoker	181 (56)	36 (57)	43 (55)	94 (55)
Non-smoker	98 (30)	19 (30)	25 (32)	51 (30)
Missing	16 (5)	2 (3)	5 (6)	9 (5)
Alcohol consumption				
Never	59 (18)	14 (22)	14 (18)	28 (16)
Former drinker	14 (4)	1 (2)	4 (5)	8 (5)
Yes	234 (72)	46 (73)	57 (73)	123 (72)
Mean intake (glasses per week) (SD)	10 (9)	7.3 (6)	9.3 (10)	10.6 (10)
Missing	19 (6)	2 (3)	3 (4)	12 (7)
BMI at diagnosis (kg/m^2^)(mean ± SD)*	26.6 (4.1)	27.5 (4.0)	26.3 (4.5)	26.5 (3.9)
Underweight	24 (7)	4 (6)	10 (13)	8 (5)
Normal weight	127 (39)	23 (37)	31 (40)	69 (40)
Overweight	113 (35)	19 (30)	19 (24)	69 (40)
Obese	58 (18)	17 (27)	16 (21)	23 (14)
Missing	4 (1)	0 (0)	2 (3)	2 (1)
Weight change before diagnosis				
> 5% weight loss	63 (19)	5 (8)	14 (18)	54 (32)
> 0 to ≤ 5% weight loss	66 (20)	15 (24)	17 (22)	62 (36)
Stable weight	181 (56)	41 (65)	42 (54)	93 (54)
Weight gain	14 (4)	1 (2)	5 (6)	6 (4)
Missing	4 (1)	1 (2)	0 (0)	3 (2)
Comorbidities				
0	68 (21)	15 (24)	20 (26)	31 (18)
1	91 (28)	21 (33)	19 (24)	48 (28)
≥ 2	166 (51)	27 (43)	39 (50)	91 (53)
Stage				
I	85 (26)	16 (25)	18 (23)	48 (28)
II	78 (24)	16 (25)	13 (17)	43 (25)
III	92 (28)	15 (24)	28 (36)	48 (28)
IV	10 (3)	1 (2)	5 (6)	4 (2)
Missing^&^	61 (19)	15 (24)	14 (18)	28 (16)
Tumour location				
Colon	222 (68%)	39 (62)	55 (71)	119 (70)
Rectum/rectum sigmoid	76 (23%)	15 (24)	18 (23)	40 (24)
Missing^&^	28 (9%)	9 (14)	5 (6)	12 (7)

^&^Cancer registry is not yet complete, so these respondents are not registered yet. *p < 0.05 between the three groups of beliefs

**Table 2:** Information provision and beliefs on the influence of nutrition of the total group of respondents (*n* = 326) and split in three groups based on the score for “belief that nutrition influences feelings of well-being”

	Respondents (*n* = 326)	Score 0–2 No belief influence (*n* = 63)	Score 3–6 Intermediate belief influence (*n* = 78)	Score 7–10 Strong belief influence (*n* = 171)
	*n* (%)	*n* (%)	*n* (%)	*n* (%)
Information provision*				
Yes	201 (62)	24 (38)	56 (72)	119 (70)
No	125 (38)	39 (62)	22 (28)	52 (30)
Number of health professionals*				
0	125 (38)	39 (62)	22 (28)	52 (30)
1	83 (26)	13 (21)	23 (30)	46 (27)
2	75 (23)	7 (11)	20 (26)	47 (28)
3	43 (13)	4 (6)	13 (17)	26 (15)
Beliefs recovery after treatment*				
0–2	74 (24)	49 (78)	13 (17)	12 (7)
3–6	69 (22)	10 (16)	36 (46)	23 (14)
7–10	169 (54)	4 (6)	29 (37)	135 (79)
Beliefs recurrence of cancer*				
0–2	123 (40)	53 (84)	27 (35)	42 (25)
3–6	113 (36)	9 (14)	38 (49)	66 (39)
7–10	75 (24)	1 (2)	13 (17)	60 (36)
Beliefs complaints*				
0–2	133 (43)	61 (97)	29 (37)	43 (25)
3–6	92 (30)	2 (3)	43 (55)	47 (28)
7–10	85 (27)	0 (0)	6 (8)	79 (47)

*p < 0.05 between the three groups of beliefs

Furthermore, the association between the kind and number of health professionals and the strength of beliefs was investigated.

For the different health professionals, the Likert scale option ‘none’ was recoded into received information ‘no’ and a little/some/a lot into ‘yes’. Usual body weight and body weight and height at the moment of diagnosis were used to calculate weight change before diagnosis and body mass index (BMI) at diagnosis. For survivors < 70 years old, a BMI 20–25 was considered a healthy BMI, for survivors ≥70 years old, a BMI 22–28 was considered a healthy BMI [23, 24]. The Global Leadership Initiative on Malnutrition (GLIM) states for survivors > 70 years old, a BMI < 22 is a low body mass index, which results in a higher risk for a mild to moderate deficit in muscle mass [23]. BMI 22.01–28 corresponded to not undernourished in people aged > 70, as stated by the team of the SNAQ RC [24].

To evaluate the association between the dependent variables having strong beliefs on the influence of nutrition on feelings of well-being (yes/no), complaints after treatment (yes/no), recovery after treatment (yes/no) and cancer recurrence (yes/no), and (1) having received nutritional information (yes/no), and (2) the number of health professionals providing nutritional information, prevalence ratios (PRs) and 95% confidence intervals were calculated. Cox proportional hazard regression analysis was used with the time variable set at 1 for each respondent. Having a strong belief corresponded to a score of 7–10 (scale 0–10) and having no strong belief corresponded to a score of 0–2 (scale 0–10). Analyses were adjusted for age and gender. Educational level (elementary school/high school; vocational education; bachelor degree or higher), smoking status (current, former or non-smoker), comorbidities (0, 1 or ≥ 2), stage (I, II, III or IV) and BMI at diagnosis (underweight, normal weight, overweight or obese) were evaluated as possible confounding factors and were included if they changed the PR by at least 10%. For strong beliefs that nutrition influences complaints and recurrence, cancer stage changed the PR with > 10%. Therefore, the Cox proportional hazard regression analyses were adjusted for age, gender and cancer stage.

Foods that respondents believed either had a positive or negative influence on their cancer were categorized.

Analyses were performed using SPSS (version 23) and *p* < 0.05 was considered statistically significant.

## Results

As can be seen in Table 1, the oldest respondents and respondents with a lower level of education had the least belief on the influence of nutrition on feelings of well-being. Respondents with a high intake of alcohol, respondents with the highest levels of weight loss and with the most comorbidities had the strongest belief. Respondents with a strong belief on the influence of nutrition on feelings of well-being had more often received information from one or more health professionals (Table 2) and were less often obese than survivors who believed there was no influence (Table 1).

A total of 125 respondents (38%) did not receive information about nutrition from their healthcare professionals (Table 2). Of the respondents who received information about nutrition (*n* = 201, 62%), 41% received information from one health professional (41% from a nurse, 36% from a dietician and 23% from a doctor), 37% of two health professionals (72% from a doctor and nurse, 11% from a doctor and dietician and 17% from a nurse and dietician) and 21% of three health professionals. No differences were seen in ratings of the different beliefs and whether the information was provided by a doctor, a nurse or a dietician.

**Fig. 1:**
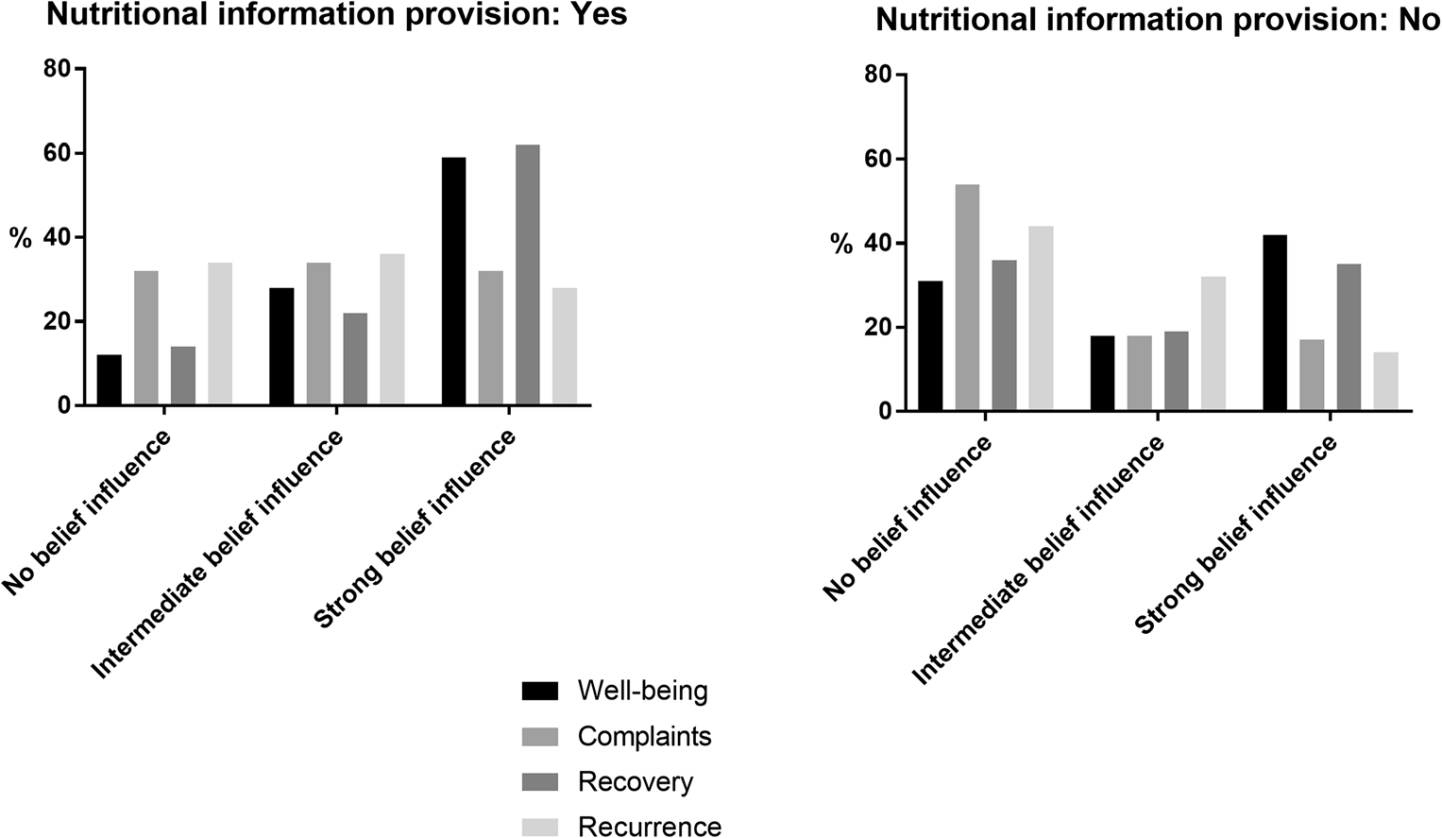
Nutritional information provision (yes/no) and beliefs that nutrition can influence feelings of well-being, complaints, recovery after treatment and recurrence of cancer

Respondents, who received nutritional information from a health professional, had stronger beliefs on the feelings of well-being, the influence of nutrition on recurrence of cancer, recovery after treatment and complaints, compared with respondents who received no nutritional information (Fig. 1 and Table 3). People, who had strong beliefs on the influence of nutrition on well-being, also had strong beliefs about the influence of nutrition on the recovery after treatment, recurrence after cancer and complaints.

**Table 3:** Association between having strong beliefs and having received nutritional information or the number of health professionals providing nutritional information

		Prevalence ratio’s (95% CI) of having a strong belief that nutrition influences			
		Well-being	Complaints	Recovery after treatment	Recurrence
Received nutritional information	No	(1)	(1)	(1)	(1)
	Yes	1.5 (1.1–2.2)	2.6 (1.4–4.7)	1.7 (1.1–2.5)	1.97 (1.00–3.89)
Number of health professionals providing nutritional information	0	(1)	(1)	(1)	(1)
	1	1.4 (0.9–2.2)	2.5 (1.3–5.0)	1.5 (0.9–2.4)	1.6 (0.7–3.6)
	2	1.57 (1.01–2.44)	2.2 (1.1–4.5)	1.67 (1.05–2.67)	1.7 (0.8–3.9)
	3	1.61 (0.97–2.68)	3.4 (1.6–7.4)	2.0 (1.2–3.3)	2.8 (1.3–6.2)

All adjusted for age, gender and cancer stage

**Fig. 2:**
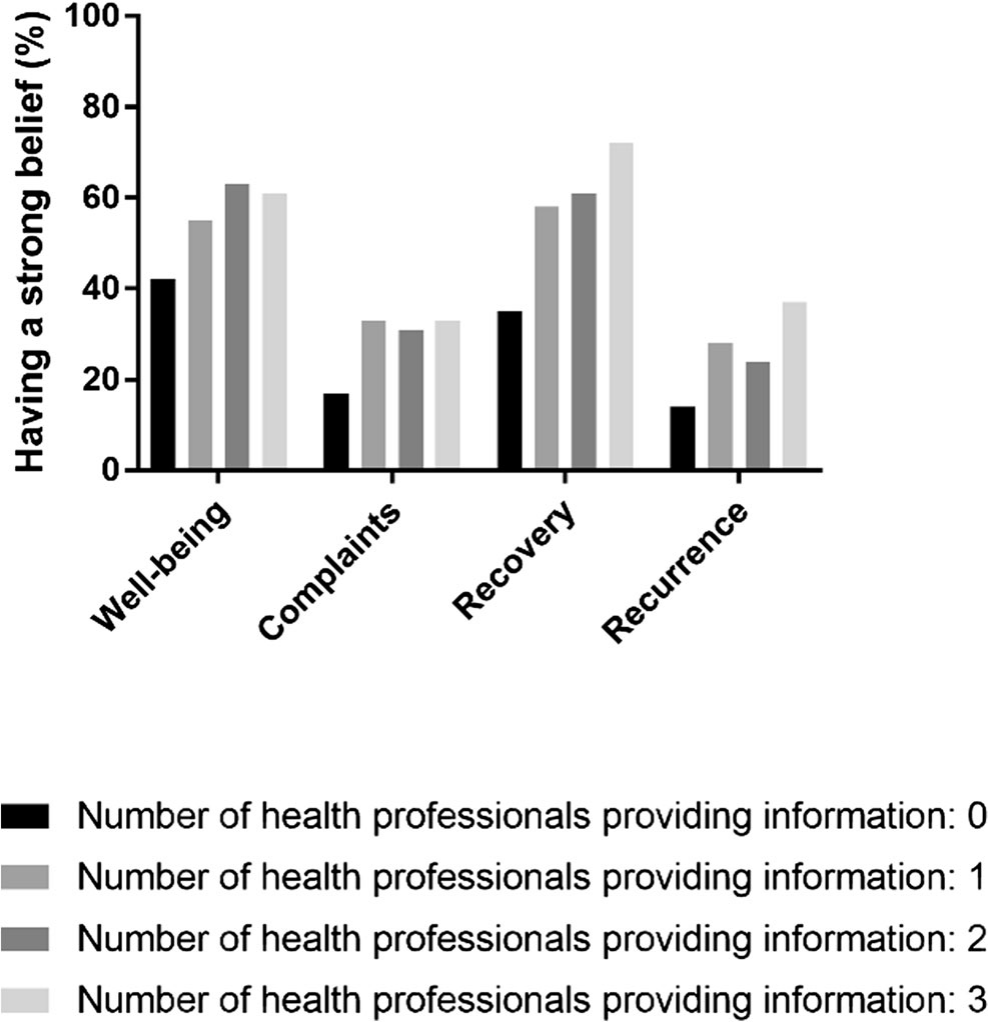
The number of health professionals providing information and the percentage of respondents having a strong belief

People, who received no information from health professionals, had the least beliefs nutrition influences well-being, complaints, recovery after treatment or recurrence. People, who received information from more health professionals, had stronger beliefs (Fig. 2 and Table 3).

Ninety-one respondents (28%) believe there are nutrients and diets that can positively influence the course of the disease the CRC. Nutrients and diets mentioned by respondents to have a positive influence were a healthy diet with plenty of fruits and vegetables (*n* = 43), fibres (*n* = 10), supplements (*n* = 9), curcumin (*n* = 8), protein-rich foods (*n* = 7), fresh products (*n* = 4), cannabidiol (*n* = 3), little meat (*n* = 2) and a diet that influences the immune system (*n* = 2).

A total of 114 respondents (35%) believe there are nutrients and diets that can negatively influence the disease. Nutrients and diets mentioned by respondents to have a negative influence were too much fat (*n* = 37), red and processed meat (*n* = 31), alcohol (*n* = 24), sugar (*n* = 20), additives (*n* = 8), burned foods (*n* = 6), processed foods (*n* = 6), salt (*n* = 5), an unhealthy diet (*n* = 4), fibre (*n* = 1), protein (*n* = 1) and vitamins (*n* = 1) (data not shown).

## Discussion

Most respondents who received information strongly believe nutrition influences feelings of well-being and recovery after treatment. No differences were found in the ratings of the different beliefs and whether the information was provided by a doctor, a nurse or a dietician. Nevertheless, it did matter how many health professionals provided nutritional information: survivors who received information from three health professionals had more often strong beliefs than those who received information from one health professional.

Weaver et al. [25] described that experiments in psychology showed that an opinion is likely to be more widely shared the more different group members express it. Participants had stronger beliefs when the same opinion was expressed once by each of three different group members than when it was expressed once by one group member [25]. In a previous study of our research group, we found that the preferred way of receiving information in a group of cancer survivors was from multiple health professionals: (oncology) nurses, dieticians and doctors, at four or more times [26]. The wish for repeated information provided by different health professionals as expressed in the previous study matches the association found in the current study. Since 59% of respondents received information from two or more health professionals in the present study, it is important to provide uniform information, to have a maximal effect of repetition, as is also supported by Weaver et al. [25].

No association was seen between the strength of beliefs and the kind of health professional who provided the information. No previous literature was found on this association. In a best-worst discrete choice experiment by Wright et al., CRC survivors expressed the wish to receive dietary information in a hospital by a bowel cancer nurse, which was preferred beyond information from a dietician or a general nurse [27]. In a survey among 175 CRC survivors, 93% indicated they wanted a conversation with their doctor about survivorship information. Sixty-six percent had received information about diet and exercise to keep them healthy, and of these people 94% found the information useful [28]. Focus groups with CRC survivors showed that they wish to receive lifestyle support in hospital, offered by a gastro-intestinal oncology nurse, an oncology dietician and/or a stoma nurse specialist. Oncologists were also mentioned to be suitable to offer or to refer to lifestyle support [29]. A survey held among young cancer survivors (mean age 20 years) showed the preferred sources of dietary information were websites and health professionals, without mentioning what kind of health professional [30]. In The Netherlands, every cancer survivor meets with the doctor and oncology nurse. Nutritional counselling by a dietician is only possible after referral.

The beliefs on foods that can positively or negatively influence the disease are mostly correct. There is indeed evidence that a diet rich in fruits, vegetables and fibres can positively influence cancer outcomes, and too much fat, red and processed meat, alcohol, much sugar, burned foods and an unhealthy diet may negatively influence the risk of cancer recurrence [31]. However, supplements, curcumin and cannabidiol do not positively influence the disease to our current knowledge. The use of supplements during chemotherapy or radiotherapy may even be counter-effective, since anti-oxidants may counteract the oxidative effect of chemotherapy and radiotherapy [32, 33].

A large part of the present study population is overweight or obese at diagnosis, which is in line with other studies [5], who also show that weight gain during and after cancer treatment in specific cancer types (e.g. breast and colorectal cancer) is very common. The conventional belief that weight gain is good and weight loss is bad during and after cancer treatment may not be in place [34].

The present study is one of the first studies investigating the association between nutritional information provision and patients’ beliefs on nutrition and cancer. Previous studies often focused on a broad range of topics on information provision to cancer survivors, such as environmental pollution, stress [35, 36], but not on patients’ beliefs on nutrition in association with information provision. Major strengths of our study are the link with the Netherlands Cancer Registry and the structured way of sending out questionnaires by the PROFILES registry. In this way, clinical information can be extracted from the Cancer Registry, instead of having to ask for this information in a questionnaire, the latter being more prone to errors. The assessment of information provision by different health professionals is another strength, not focusing on one type of health professional.

There were also some limitations. Due to the short time between the questionnaire and the writing of this manuscript, not all clinical data was registered in the Netherlands Cancer Registry. There is always a delay between the diagnosis of cancer and appearance in the Netherlands Cancer Registry. In the current study, this led to a number of missing values in tumour location and stage. Second, survivors have been actively recruited by oncology nurses and could not be recruited if they were already participating in another study. This may have led to the inclusion of a specific survivor population, because of the inclusion criteria of the other studies. The age and sex distribution of our study population indeed shows that we have a specific survivor population, since our population included more men (61% vs. 56%) and younger patients (59% vs. 50% < 70 years old) compared with the Netherlands Cancer Registry population of 2016 [2]. Recruitment took place in four hospitals, which is a selection of peripheral hospitals in the region. A third limitation is that the authors did not know what kind of nutritional information was given to the cancer survivors by the health professionals. It might be possible that not all advice given was according to the latest insights and knowledge. Other studies did report about the provided advice: participants reported to have received the advice to gain weight by eating whatever they liked, and that they were not discouraged to eat unhealthier foods. This was in contrast with the advice that should have been provided in this study. Also, exercise was not encouraged by nurses in this study, while this was part of the study programme. Patients with a stoma had been told to eat bland low-fibre foods, which is not in line with the dietary guidelines [6, 37, 38].

Another limitation is that our study was based on cross-sectional data, so nothing can be concluded about causal relationships between the provision of nutritional information and changes in beliefs on the effect of nutrition on cancer recurrence, recovery, feelings of well-being and complaints. A last limitation is the relatively short study duration, of 4 weeks. In this timeframe, shortly after diagnosis, most advice will be on surgical recovery, on nutritional needs related to a stoma and digestion problems [37]. In the weeks and months after the completion of acute treatments, nutritional needs may include advice on protein intake, weight loss or weight gain, diarrhoea, xerostomia, anorexia and food aversion [6].

## Conclusion and recommendations

The current study shows that it is important for cancer survivors to receive information about nutrition and cancer, since it might positively influence cancer survivors’ beliefs on the effect of nutrition on cancer recurrence, recovery, feelings of well-being and complaints. Repeating the information by different health professionals is important in strengthening correct beliefs on nutrition and cancer. The beliefs on foods that can positively or negatively influence the disease are mostly correct.

Future research should focus on whether it is more important to have the same message repeated by one health professional several times, or that the same message is spread by different health professionals. We speculate that in patient care, several health professionals should bring the same message to the patient, to confirm the information already given. This might be the solution in altering nutritional beliefs and thereby altering nutritional behaviour of CRC survivors. To make sure that every health professional brings the same message, it is important to make one person (e.g., a dietician) responsible for keeping the other health professionals up to date about evidence- or practice-based nutritional advices and changes in these advices. Furthermore, the dietician must take the lead in arranging the procedure concerning nutritional screening (when and by whom), which basic advice can be given and by whom and when patients need to be referred. All given dietary advice and used information sources must be registered in the patient file. In this way, all health professionals can refer to this information source and inform the survivor in a uniform manner.
